# Evaluation of Anti-Activated Factor X Activity and Activated Partial Thromboplastin Time Relations and Their Association with Bleeding and Thrombosis during Veno-Arterial ECMO Support: A Retrospective Study

**DOI:** 10.3390/jcm10102158

**Published:** 2021-05-17

**Authors:** Mouhamed Djahoum Moussa, Jérôme Soquet, Antoine Lamer, Julien Labreuche, Guillaume Gantois, Annabelle Dupont, Osama Abou-Arab, Natacha Rousse, Vincent Liu, Caroline Brandt, Valentin Foulon, Guillaume Leroy, Guillaume Schurtz, Emmanuel Jeanpierre, Alain Duhamel, Sophie Susen, André Vincentelli, Emmanuel Robin

**Affiliations:** 1 CHU Lille, Pôle d’Anesthésie-Réanimation, 59000 Lille, France; antoine.lamer@chru-lille.fr (A.L.); guillaume.gantois@chru-lille.fr (G.G.); vincent.liu@chru-lille.fr (V.L.); caroline.brandt@chru-lille.fr (C.B.); valentin.foulon@chru-lille.fr (V.F.); guillaume.leroy@chru-lille.fr (G.L.); emmanuel.robin@chru-lille.fr (E.R.); 2 CHU Lille, Service de Chirurgie Cardiaque, 59000 Lille, France; jerome.soquet@chru-lille.fr (J.S.); natacha.rousse@chru-lille.fr (N.R.); andre.vincentelli@chru-lille.fr (A.V.); 3 Univ. Lille, INSERM, CHU Lille, CIC-IT 1403, 59000 Lille, France; 4 Univ. Lille, CHU Lille, ULR 2694-METRICS: Évaluation des Technologies de Santé et des Pratiques Médicales, 59000 Lille, France; julien.labreuche@chru-lille.fr (J.L.); alain.duhamel@chru-lille.fr (A.D.); 5 CHU Lille, Department of Biostatistics, 59000 Lille, France; 6 CHU Lille, Pôle d’Hématologie-Transfusion, Centre de Biologie Pathologie Génétique, 59000 Lille, France; annabelle.dupont@chru-lille.fr (A.D.); emmanuel.jeanpierre@chru-lille.fr (E.J.); sophie.susen@chru-lille.fr (S.S.); 7 Department of Anesthesiology and Critical Care Medicine, Amiens University Hospital, 80054 Amiens, France; osama.abouarab@gmail.com; 8 MP3CV, EA7517, CURS, Jules Verne University of Picardie, 80054 Amiens, France; 9 CHU Lille, Pôle de Cardiologie, 59000 Lille, France; guillaume.schurtz@chru-lille.fr

**Keywords:** extracorporeal membrane oxygenation, activated partial thromboplastin time, anti-factor Xa, intravenous unfractionated heparin monitoring, thrombotic complications, bleeding

## Abstract

Background: We aimed to investigate the relationship between anti-activated Factor X (anti-FXa) and activated Partial Thromboplastin Time (aPTT), and its modulation by other haemostasis co-variables during veno-arterial extracorporeal membrane oxygenation (VA-ECMO) support. We further investigated their association with serious bleeding and thrombotic complications. Methods: This retrospective single-center study included 265 adults supported by VA-ECMO for refractory cardiogenic shock from January 2015 to June 2019. The concordance of anti-FXa and aPTT and their correlations were assessed in 1699 paired samples. Their independent associations with serious bleeding or thrombotic complications were also analysed in multivariate analysis. Results: The concordance rate of aPTT with anti-FXa values was 50.7%, with 39.3% subtherapeutic aPTT values. However, anti-FXa and aPTT remained associated (β = 0.43 (95% CI 0.4–0.45) 10^−2^ IU/mL, *p* < 0.001), with a significant modulation by several biological co-variables. There was no association between anti-FXa nor aPTT values with serious bleeding or with thrombotic complications. Conclusion: During VA-ECMO, although anti-FXa and aPTT were significantly associated, their values were highly discordant with marked sub-therapeutic aPTT values. These results should favour the use of anti-FXa. The effect of biological co-variables and the failure of anti-FXa and aPTT to predict bleeding and thrombotic complications underline the complexity of VA-ECMO-related coagulopathy.

## 1. Introduction

Veno-arterial extracorporeal membrane oxygenation (VA-ECMO) is a lifesaving therapy in refractory cardiogenic shock with an exponential increase in use over the last few decades. Despite the improvement in head-pump technologies, surface coating of tubing and the use of contemporary compact polymethylpenten membrane oxygenators, VA-ECMO remains associated with a high incidence of thrombotic and bleeding complications [1,2,3,4].

These complications increase morbidity and cost, and may also impact mortality in supported patients [4]. To prevent thrombotic events and the activation of inflammatory pathways [5,6], intravenous anticoagulation is mandatory with unfractionated heparin (UFH) as a reference treatment [7].

Despite this systematic intravenous anticoagulation, thrombotic complications occur in 20% to 30% of supported patients and, even worse, bleeding events concern up to 70% of VA-ECMO patients [2,3,4,8], questioning the assessment of the efficacy and safety of UFH treatment.

The most commonly used assays to monitor UFH under VA-ECMO are activated clotting time (ACT), activated partial thromboplastin time (aPTT) and anti-factor X activity (anti-FXa). The currently available guidelines do not recommend an assay over the other [9,10]. If ACT was shown to be irrelevant for anticoagulation monitoring in VA-ECMO as compared to anti-FXa and aPTT after the cannulation procedure [11,12], there is no clear evidence of the superiority of the anti-FXa assay over aPTT when excluding pre-analytical characteristics. Their ability to reflect the sole heparin intrinsic activity can be questioned knowing that both anti-FXa (to a lesser extent) and aPTT may be susceptible to several biological factors unrelated to UFH therapy and to analytical limits. The impact of these biological variables that included hyperbilirubinemia, hyperfibrinogenemia or reduced clotting factors, on anti-FXa and aPTT association during VA-ECMO support [13] are poorly characterised.

Furthermore, the comparative analysis of the association of anti-FXa and aPTT with bleeding events or thrombotic complications in a VA-ECMO setting is lacking. This lack of evidence may explain the concerning high discrepancy that exists among centres for anticoagulation treatment and monitoring [14,15].

Our main objectives were to study the association between anti-FXa and aPTT during VA-ECMO support, to evaluate the modulation of this association according to prothrombin time (PT), factor V, fibrinogen, bilirubin, lactate dehydrogenase (LDH) and platelets count. Besides, we sought to investigate the association of anti-FXa and aPTT with the occurrence of bleeding events and thrombotic complications.

## 2. Materials and Methods

This retrospective single-centre study was conducted at the Lille University Hospital Cardiac and Thoracic Intensive Care Unit. Approval was obtained from the Ethical Committee of French Society of Anaesthesia and Intensive Care Medicine (CERAR, IRB 00010254-2020-195), Paris, France (Chairperson Prof J.E. Bazin) on 11 April 2020 which waived the need for informed consent because of the retrospective setting. All datasets were declared to the French authorities in compliance with national laws (CNIL N° DEC2015-14).

### 2.1. Participants

Consecutive adult patients aged 18 years and older, supported by VA-ECMO for refractory cardiogenic shock between January 2015 and June 2019 were included. Exclusion criteria were moribund patients (duration of support <24 h), missing or inadequate biological factors (exposure to direct oral anticoagulants, to low molecular heparin or to thrombolysis agent at cannulation), missing outcome status, known haematological diseases (heparin induced thrombocytopenia, ADAMTS13 deficiency, antiphospholipid syndrome), and the use of argatroban as primary anticoagulation therapy. In cases of multiple VA-ECMO support, only the first run was considered.

### 2.2. Data Collection and Sources

Anthropometric, anamnestic and outcomes variables were extracted from our electronic health records (Sillage (SIB, Rennes, France) and IntelliSpace Critical Care and Anaesthesia (Philips Healthcare, Koninklijke Philips N.V. Eindhoven, The Netherlands)). Biological data were recovered from the laboratory results management software (Molis^®^, CompuGroup Medical, Koblenz, Germany).

### 2.3. Clinical Management of VA-ECMO

Extracorporeal centrifugal pump system (Rotaflow (Maquet Gentige group, Rastatt, Germany), Revolution (LivaNova Group, Saluggia, Italy), Centrimag (Thoratec, Pleasanton, CA, USA)) associated with following membrane oxygenator (Quadrox (Maquet Gentige group, Rastatt, Germany) or Eos ECMO (LivaNova Group, Saluggia, Italy) or A.LONE ECMO oxygenator (Euroset, Medolla, Italy)) were used. All VA-ECMO circuits were primed with crystalloids at the initiation of support and in cases of circuit change.

Senior cardiac surgeons performed all cannulation either for peripheral (femoral vein and femoral or subclavian arteries) or central VA-ECMO (right atrium and ascending aorta or pulmonary artery). Left ventricle (LV) unloading was considered in case of LV dilatation with spontaneous contrast in cardiac ultrasound or pulmonary oedema, using Impella CP or 5.0 (Abiomed, Inc, Danvers, MA, USA), additional LV venting cannulation, cannulation change for anterograde flow or intra-aortic balloon pump. The pump flow was adjusted to target a mean arterial pressure >60 mmHg, SvO2 > 65% or ScVO2 > 70%, aortic valve opening and optimal right ventricle unloading. Weaning was considered for recovery when cardiac output was acceptable (aortic or pulmonary velocity time integral >12 cm/s or 10 cm/s respectively) after lowering the ECMO flow with no or reduced inotropic support.

### 2.4. Anticoagulation and Bleeding Management

We use anti-FXa as the primary assay to guide UFH treatment in our ICU. UFH bolus of 100 IU/kg was initiated just before cannulations. Intravenous UFH was administered in ICU and adjusted to obtain an anti-FXa of 0.2 to 0.4 IU/mL. This target was increased to 0.4 to 0.7 IU/mL if pump flow <1.8 L/min or in case of thrombotic complications. Of note UFH could be postponed after cannulations (VA-ECMO following cardiopulmonary bypass) or stopped until bleeding was managed at the physician’s discretion. Before ICU admission, patient haemostasis was managed during surgery (postcardiotomy patients) using ACT (targeting 200 s to 250 s before cannulation), and/or aPTT, PT and fibrinogen and more recently thromboelastography (2019). For non-postcardiotomy patients, ACT was used at the ECMO cannulations only.

During ICU stay, packed red blood cells (PRBC) and platelet concentrates were transfused to maintain a haemoglobin level >8 g/dL and platelet count >50,000 mm^−3^ (increased to 70,000 mm^−3^ in case of bleeding). Fresh frozen plasma (FFP) was given in case of major overt bleeding that required massive transfusion with a high FFP: RBC ratio or according to multi-parametric analyses of coagulation tests (PT, aPTT, factor V, factor II, fibrinogen and thromboelastographic test). Antithrombin was measured when heparin resistance was suspected (UFH dosage >10 IU/kg/h for 0.2 IU/mL to 0.4 IU/mL target) and supplementation (Aclotine, LFB Biomedicaments, Les Ulis, France) was performed when the level was <50%.

### 2.5. Sampling and Laboratory Assays

Samples were drawn from the patient through indwelling arterial line, or central venous catheter, and sent to the haemostasis and biology department using an automatised pneumatic tube transport system which shortens the laboratory delivery time to a few minutes. All haemostasis variables (aPTT, anti-FXa, PT, fibrinogen, factor V) were measured on the same sample at each time point. Sampling for platelet count, total bilirubin and LDH were drawn at the time of haemostatic sampling.

Blood samples for haemostasis tests were drawn in a 2.7 mL volume and 3.2% citrated tubes (BD Vacutainer^®^, Plymouth, PL6BP; United Kingdom) and analysed on STA-R Max analyser (Diagnostica Stago, Asnière, France). aPTT and anti-FXa measurements were performed using the same reagents during the study period. Collected samples were processed within 10 min and results given within an hour. Both measurements were performed on platelet-poor plasma (platelet count <10,000 mm^−3^) obtained after a double centrifugation at 2500× *g* for 15 min at room temperature using a chromogenic method without correction for antithrombin for anti-FXa (Biophen Heparin LRT, HYPHEN BioMed, Neuville-sur-Oise, France), and a chronometric method for aPTT (TriniCLOT aPTT HS, Tcoag, Wicklow, Ireland).

Full description of the analysers, reagent, normal value ranges or reference values of laboratory tests studied are provided in Table S1.

### 2.6. Description of aPTT and Anti-FXa Variables

aPTT and anti-FXa were measured 3 times a day with additional measurements 4 to 6 h after any change in UFH dose or in the 2 h following plasma products transfusion. To account for diurnal variation of anti-FXa and aPTT, we selected the measurement performed between 4 and 6 am, [16] which was concomitant to the measurement of the other haemostatic covariates (factor V, fibrinogen, PT, platelets) and biochemical co-variables (bilirubin and LDH). These variables are defined as reference with an “r” attached as a prefix (i.e., r_aPTT and r_anti-FXa) and were exclusively used to analyse the relationship between anti-FXa and aPTT.

Besides, we calculated daily maximum (max_aPTT, max_anti-FXa, max_fibrinogen, max_platelet), minimum (min_aPTT, min_anti-FXa, min_fibrinogen, min_platelet) and mean (mean_aPTT, mean_anti-FXa, mean_fibrinogen, mean_platelet) values for anti-FXa, aPTT fibrinogen and platelets. We used these variables in the outcome prediction analyses. All variables were recorded for 10 days following cannulation or less in case of ECMO weaning or death.

### 2.7. Study Endpoints

The efficacy endpoint of this study was thrombotic complications and the safety endpoint was serious bleeding. The thrombotic complications endpoint was a composite of stroke, limb ischaemia and ECMO circuit changes for thrombosis or cannulation thrombosis or any thrombosis that led to medical or surgical intervention or death. Major bleeding was defined according to the ELSO bleeding management guidelines [9] as a bleeding that led to surgical exploration or characterised by its location (central nervous system, hemothorax, retro-peritoneal bleeding) or requiring immediate transfusion of at least 2 units of PRBC for either a sudden fall of haemoglobin of 2 g/dL in less than 24 h or new hemodynamic instability or overt bleeding.

### 2.8. Statistical Analysis

The statistical analysis plan was approved by the authors before analyses began. Quantitative variables were expressed as means (standard deviation) for normally distributed variables or as medians (interquartile range) if otherwise. The normality of distributions was assessed using histograms and the Shapiro–Wilk test. Categorical variables were expressed as numbers (percentage). We estimated the cumulative incidence of serious bleeding and thrombotic complications during ECMO duration using the Kalbfleisch and Prentice method [17] by taking into account death under ECMO and ECMO weaning as competing events.

The first part of the analysis consisted in assessing the relationship between r_anti-FXa and r_aPTT. Following the clinical laboratory standards institute and other guidelines, we determined ex vivo the aPTT range corresponding to the anti-FXa range of 0.30 to 0.70 IU/mL [18,19]. This expected range was 62 to 109 s in our centre. When the observed r_aPTT values felt into this expected range, they were considered concordant with the r_anti-FXa range of 0.30 to 0.70 IU/mL.

We also estimated the slope between r_anti-FXa and r_aPTT values by using mixed linear regression model (unstructured covariance pattern model) to account correlation for samples drawn within the same patients considering r_anti-FXa as dependent variable, and r_aPTT as fixed independent variables. We assessed the relationship between r_anti-FXa and r_aPTT values according to other biological levels (prothrombin time, fibrinogen, factor V, platelets count, bilirubin and LDH) categorised according to quartiles using linear mixed regression models including the corresponding interaction term as fixed effects.

The second part of analysis consisted in the assessment of the relationship between the daily measures (maximum, minimum and mean daily values) of anti-FXa and aPTT and the incidence of serious bleeding and thrombotic complications under ECMO. We used a cause-specific Cox’s regression models by considering death under ECMO and ECMO weaning as competing risk, and by treating biological data (as continuous or as categorical variables according to quartiles) before outcome occurrence as time dependent co-variables to account for changes in biological values over time [20]. Since we could not ascertain that day 0 biological data were measured before the bleeding and thrombotic events that occurred that same day, we carried out a landmark analysis from day 1. Multivariable cause-specific Cox’s regression models were performed to adjust for predefined confounders (age, body mass index, SAPS II, postcardiotomy aetiology, left ventricle unloading, and daily measures of fibrinogen and platelets). All statistical tests were performed at the two-tailed α level of 0.05. Data were analysed using the SAS software package, release 9.4 (SAS Institute, Cary, NC, USA).

## 3. Results

### 3.1. Study Population

Of the 379 patients screened during the study period, 265 patients met the inclusion criteria. The study flowchart is shown in Figure S1. Patient demographics, clinical characteristics and VA-ECMO course are summarised in Table 1.

### 3.2. Relationship between Anti-FXa and aPTT Using Reference Samples

**Figure 1:**
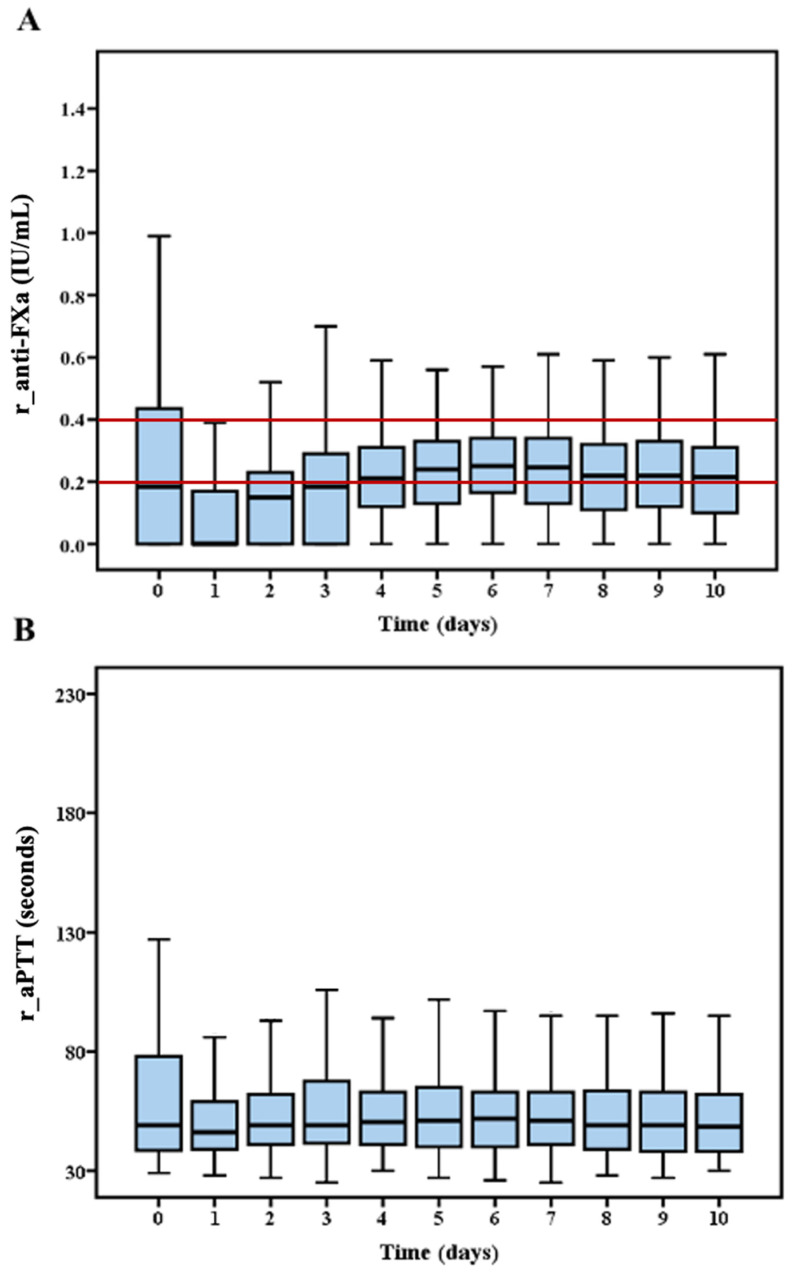
Time courses of reference anti-FXa and aPTT. Tuckey diagrams showing the daily values of reference anti-FXa (**A**) and reference aPTT (**B**) used for the association analysis. Anti-FXa was the reference test for anticoagulation monitoring and the red lines (**A**) describe the targeted anti-FXa interval.

A total of 1699 reference samples from 245 patients were used to analyse the relationship between r_anti-FXa and r_aPTT. r_anti-FXa met the clinical target of 0.2 to 0.4 IU/mL on all days except day 1 to day 3. The time courses of both variables are shown in Figure 1.

**Figure 2:**
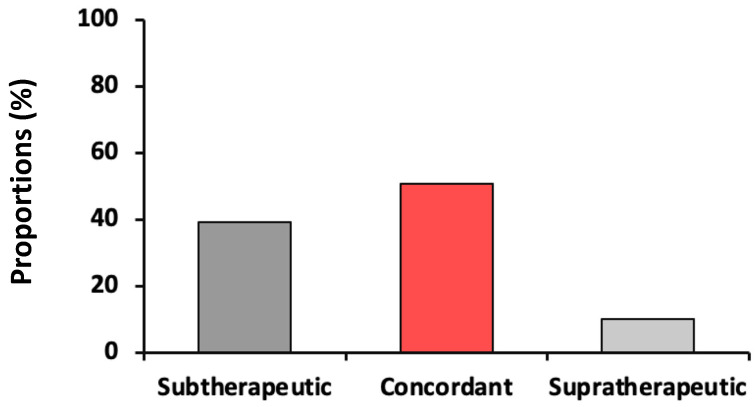
Description of concordance and discordance of anti-FXa and aPTT. Both variables were measured on the same sample. Observed aPTT values were considered concordant if they felt into the expected aPTT range that corresponds to the anti-FXa range of 0.3 to 0.7 IU/mL.

The concordance of actual r_aPTT values with expected aPTT range that corresponds to r_anti-FXa range of [0.30 to 0.70 IU/mL] was 50.7%. Expected r_aPTT values were markedly sub-therapeutic (39.3%) (Figure 2).

**Figure 3:**
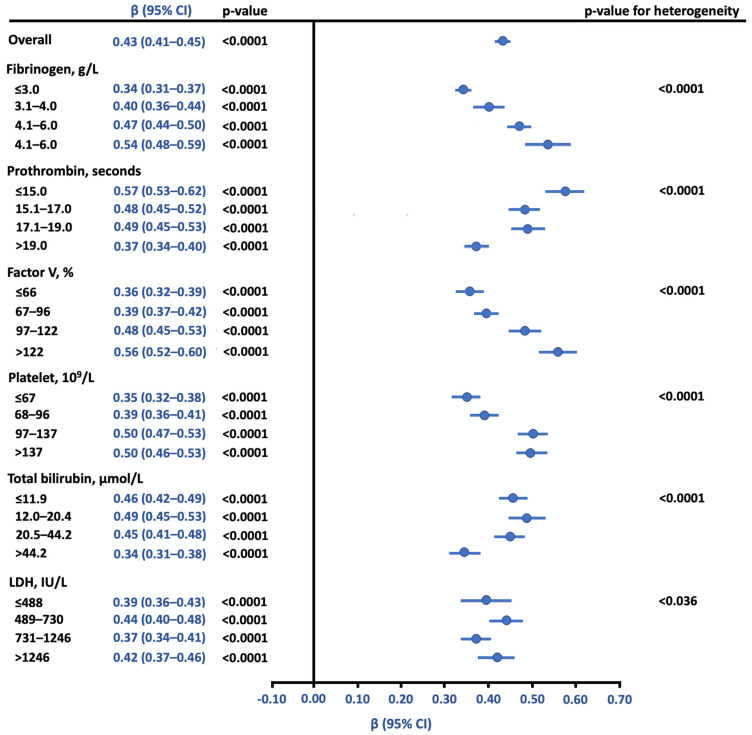
Association of anti-FXa and aPTT, overall and after modulation by biological covariates. β indicates regression coefficient of aPTT on anti-FXa (expressed as 10^−2^ IU/mL) calculated using all available biological measures at the same time points by using linear mixed model (unstructured covariance pattern model to account repeated measurements). Subgroups analyses on other biological data (categorised according to quartiles of all available measures) were made by including the corresponding interaction term into linear mixed models. Prothrombin time (PT), factor V and fibrinogen were analysed on the same citrated samples as aPTT and Anti-FXa. Platelets, bilirubin and lactate dehydrogenase samples were drawn at the same time point as aPTT and Anti-FXa.

In a mixed linear regression model, anti-FXa was associated with aPTT, with an estimated regression coefficient of 0.43 10^−2^ IU/mL (95% CI, 0.41 to 0.45) per 1 s increase in aPTT, *p* < 0.001. As shown in Figure 3, this relationship was significantly modulated by all co-variables (the time courses are shown in Figure S2). Increase in fibrinogen, factor V, and platelet levels strengthened this association whereas increase in PT and bilirubin weakened the association.

### 3.3. Association of Anti-FXa and aPTT with Serious Bleeding

**Figure 4:**
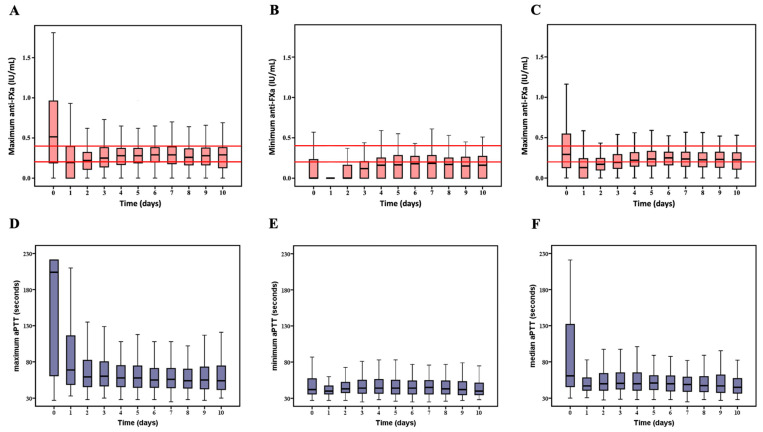
Time courses of daily maximum, minimum and mean values of anti-FXa and aPTT. The pink Tuckey diagrams showing the time course of the daily maximum (**A**) minimum (**B**) and mean (**C**) values of anti-FXa. The reference line for the lower and the upper limit of the anti-FXa used in our centre are coloured red. The bleu Tuckey diagrams showing maximum (**D**), minimum (**E**) and mean (**F**) values of aPTT.

Overall, 150 (56.6%) patients had one or more episodes of serious bleeding (Table 1), most events occurring within the first 7 days of ECMO support. The cumulative incidence of bleeding at 7 and 14 days were 53.6% and 56.2%, respectively (Figure S3). The time courses of daily anti-FXa and aPTT variables are shown in Figure 4.

In a time-dependent cause-specific Cox’s proportional hazard model, daily maximum, minimum and mean anti-FXa and aPTT values were not associated with serious bleeding before or after pre-specified adjustment on age, BMI, post-cardiotomy shock, SAPS-II score, LV unloading and daily measure of fibrinogen and platelets (Figure S4).

### 3.4. Association of Anti-FXa and aPTT with Thrombotic Complications

The cumulative incidence of thrombotic complications during ECMO support at 7 and 14 days were 25.7% and 29.8%, respectively (Additional Figure 3). Overall, 87 (32.8%) patients had one or more thrombotic complication during ECMO support (Table 1). As shown in Figure S4, thrombotic complications were not significantly associated with daily maximum, minimum or mean anti-FXa and aPTT values.

## 4. Discussion

This study is, to our knowledge, the first to examine the clinical uncertainty regarding the use of anti-FXa instead of aPTT in adult patients supported by VA-ECMO. We found a low concordance rate between r_anti-FXa and r_aPTT for the reference anti-FXa targets of 0.30 to 0.70 IU/mL. Discordant values were mainly sub-therapeutic r_aPTT values (39.3%). Although a significant association of r_anti-FXa with r_aPTT values was observed, this association was modulated by several biological variables for which abnormal values are frequently reached in VA-ECMO setting. Finally, the maximum, minimum and mean values of both anti-FXa and aPTT failed to predict serious bleeding or thrombotic complications.

The poor concordance rate (50.7%) between anti-FXa and aPTT observed in the anti-FXa target of 0.30–0.70 IU/mL in our study is in line with prior observations, where these rates varied from 35% to 60% [21,22]. However, the single study that investigated this issue in 24 patients under mechanical circulatory support (HeartMate II, Thoratec Corporation, Pleasanton, CA, USA) observed a worse concordance rate (32.1%) in comparison to our results for the anti-FXa therapeutic target of 0.30 to 0.70 IU/mL.

Differences in aPTT reagent/analyser performances could partially explain this result [23], as could the effects of laboratory co-variables on the association between anti-FXa and aPTT shown by our results.

The observed modulation of this association by bilirubin levels was previously reported in vitro and in paediatric patients supported by ECMO [14]. We suspected and confirmed the interaction of factor V—which may directly alter aPTT—with the association between anti-FXa and aPTT. The ability of fibrinogen to modulate aPTT [13,23] can explain why this variable could also alter the association between anti-FXa and aPTT. Intriguingly, platelet count also affects the anti-FXa and aPTT relationship while their measurement is performed on centrifuged plasma without taking platelet contribution to clot formation into account. This observation suggests that platelet components with a link to the total platelet count (beyond the usual residual platelet in platelet-poor plasma) may be involved in modulating the anti-FXa and aPTT interaction. In this hypothesis, platelet microparticles (MPs) and platelet fragments can be evoked. Indeed, these platelet MPs have a pro-thrombotic ability and could, therefore, alter the reliability of the measurement of aPTT values. The presence of these MPs has already been established in a paediatric ECMO model [24]. They could be generated following the shear-induced platelet activation mediated by the glycoprotein (GP) Ib α-Von Willebrand factor (VWF) interaction [25,26] or by a direct traumatic effect of high shear stress under VA-ECMO [27,28]. Although the association between platelet count—which was actually found to modulate the anti-FXa/aPTT interaction in our multivariate model—and the effect of MPs is difficult to establish, some data support this hypothesis. The thrombocytopenia observed under ECMO is in part the result of apoptosis induced by high shear stress, yet this same trigger is at the origin of platelet MPs generation [25,29,30].

Of concern is the fact that these biological co-variables varied over time in different directions, adding more complexity in the way anti-FXa and aPTT association is modulated.

As aPTT encompasses the loss of coagulation factors of intrinsic pathway from Factor XII and its activators (prekallikrein and high-molecular-weight kininogen) to fibrinogen, it might be assumed that this variable is more reliable than anti-FXa in predicting bleeding events. We have analysed this hypothesis by studying the association between maximum, minimum and mean values of aPTT and anti-FXa with haemostatic complications but no significant association was observed.

Similar results were observed by Mazzeffi et al. in a retrospective analysis of 132 patients extracted from their institutional records and ELSO database [31]. Conversely, these results contradicted those of Aubron et al. for bleeding outcomes. These authors studied a mixed population of veno-venous and VA-ECMO patients and found a significant association of aPTT value >70 s with bleeding [4]. Concerning thrombotic complications and ECMO, all these studies and our findings clearly converged to confirm the lack of statistical interaction between aPTT and thrombotic complications [4,31]. Only one study in a paediatric ECMO setting reported an association between anti-FXa and ECMO circuit change [11]. In a recent multicentric retrospective study, a mean anti-FXa >0.46 IU/mL was found to be associated with serious bleeding in a mixed population of medical VA-ECMO and VV-ECMO patients. This result triggered our investigation in the specific population of VA-ECMO patients including both post-cardiotomy and medical ECMO [32]. Differences in study population, in statistical approaches, variables and outcome definitions may have played a role in these opposite results.

Our results, therefore, suggest that bleeding complications are the result of a multifactorial process that goes beyond the administration of UFH or anti-FXa or APTT values. Indeed, surgical insult, anaemia, thrombocytopenia, fibrinogen concentration, or acquired von Willebrand syndrome (almost ubiquitous under ECMO), certainly influence the risk of bleeding [33]. Regarding thrombotic complications, ECMO devices and cannula characteristics, the levels of pro-thrombotic biological variables and haemolysis (which may trigger inflammation and thrombosis) are to be accounted for [34]. To a lesser extent, flow induced platelets activation and aggregation should also be considered [6,35]. Furthermore, pre-analytical and biological confounders described above may result in falsely increased or reduced values and may blur the hypothetical contribution of anti-FXa and aPTT to this prediction.

Based on our results and considering the evidence available in the literature, we propose the use of anti-FXa rather than aPTT, bearing in mind that anti-FXa remains the least used test for monitoring UFH under ECMO worldwide [15]. First, we observed the highest rate of sub-therapeutic aPTT levels (39.3%) in the corresponding anti-FXa therapeutic range most used worldwide [15] during ECMO support, suggesting that the use of aPTT for UFH monitoring may result in increased UFH infusion in almost 40% of patients. Second, compared to anti-FXa, aPTT is more sensitive to pre-analytical considerations such as under-filled blood sample tubes, diurnal fluctuations, acute phase reactants, intrinsic or common pathway factors levels and antiphospholipid antibodies [13,23,36]. Additionally, variations in reagents and laboratory specificities are more pronounced with aPTT [22]. Indeed, unlike PT which is normalised between centres using the International Normalised Ratio, each laboratory must set up a specific heparin therapeutic range, resulting in serious discordance between centres [23,37].

Our study has several strengths and suffers from several limitations. In this large number of paired samples analysed, we used a comprehensive statistical approach that took into account repeated measurements and multiple comparisons, the time effect and the time-dependent nature of the studied variables. Similarly, the outcome prediction models included competing events that may increase studied outcomes regardless of the effect of the studied variables.

The retrospective setting of our study is likely to favour several biases among which misclassifications of bleeding and thrombotic events are the more prominent, particularly in sedated patients. The single-centre design, which may represent a limit for external validity for outcome prediction, can be considered as a strength for the analyses of anti-FXa and aPTT relationship. Assays’ and reagents’ sensitivity to heparin use for aPTT and to a lesser extent for anti-FXa may be different among centres. In addition, even with a similar assay/regent, inter-laboratory agreement in the monitoring of UFH using aPTT was proven to be a serious source of concern [23,38]. Moreover, pre-analytical processes may vary widely between centres. It should be noted that our anti-FXa test was sensitive to antithrombin deficiency. In the presence of this deficiency and depending on its severity, the anti-FXa measured in our study could misrepresent the actual circulating heparin concentration. In this situation of antithrombin deficiency, our anti-FXa test, nevertheless, reflects a value that really corresponds to the patient’s UFH effect on factor X. Therefore, a discrepancy between the anti-FXa value and the dose of UFH administered will indicate the need for possible antithrombin supplementation, as opposed to a test corrected for antithrombin which would be falsely reassuring.

Plasma products transfused mainly due to serious bleeding could alter the interpretation of aPTT and anti-FXa. However, this risk is rather low in our study. Indeed, for the analysis of concordance and association between anti-FXa and APTT, we only used the first measurements, performed in the morning, at a distance from any transfusion. For serious bleeding and thrombotic complications prediction analyses, the variables studied were the daily maximum, minimum and means, which may reduce the potential confounding effect of such transfusions. In addition, a control of the coagulation parameters is systematically carried out in our protocol in the hour following the end of such transfusion when they occurred.

We did not include veno-venous (VV)-ECMO patient in this study on purpose. VV-ECMO patients differ from VA-ECMO primarily by the type of insult (acute respiratory distress syndrome vs. refractory cardiogenic shock) and by various support characteristics that modulate thrombotic and bleeding complication profiles. Thrombotic complications and bleeding events occur less often in the VV-ECMO patients. Indeed, the pressure regimen in the arterial cannulas may increase the risk of bleeding. Acute limb ischaemia mainly burdens VA-ECMO patients cannulated through peripheral arteries but rarely VV-ECMO patients. Similarly, acute exposure to P2Y12 inhibitors is observed exclusively in ischemic cardiogenic shock patients. Furthermore, acute liver failure observed in cardiogenic shock that impact factor V concentration is more limited in ARDS patients under VV-ECMO support [39].

We herein provide important information regarding the limitations, reliability and relevance of anti-FXa and aPTT assays during ECMO support, both for UFH monitoring and haemostatic complications prediction. This information should be considered in the design of future prospective control trials evaluating these assays in VA-ECMO patients. Furthermore, it is crucial to evaluate alternative haemostasis tests that better consider the current knowledge about ECMO-related coagulopathy, either alone or in association.

To this end, associating platelet function tests that explored primary haemostasis with aPTT and PT may help to refine the description of a patient’s coagulation state [40]. Platelets play a pivotal role in haemostasis and are key contributors to VA-ECMO related coagulopathy. Two VA-ECMO main characteristics influence platelets homoeostasis during VA-ECMO support: the VA-ECMO artificial surface which promotes platelet adhesion to their surface and of most importance, the VA-ECMO induced high shear stress. This high shear stress leads to conformational changes in the VWF, allowing its proteolytic cleavage by the metalloprotease ADAMTS-13 into smaller but less efficient fragments, and promote GPIb α-mediated interaction between this protein and platelets. High shear stress has also been shown to cause loss of platelet surface receptors, particularly GPIb α and GPIV shedding and can trigger platelets apoptosis through GPIb α-VWF interaction [25,26,27,28,29,30]. The resulting platelet functional changes along with the platelets count loss are critical in haemostatic complications observed during VA-ECMO.

The latest generation of viscoelastic tests that explore the entire coagulation cascade, while making it possible to dissociate the effect of UFH from that of the other coagulation determinants, should be further explored in regard to the promising preliminary results available [41]. Finally, thrombin generation tests (unlike aPTT, anti-FXa or PT that only explore the coagulation initiation phase) which study the amplification phase (95% of thrombin generation), giving a more accurate reflection of the real coagulability of patients, could be an alternative [42].

## 5. Conclusions

Anti-FXa and aPTT were discordant and are thus not interchangeable for UFH monitoring in VA-ECMO setting. The association between both assays was strongly modulated by several biological cofactors which pathological values frequently observed under ECMO. Therefore, these variables should be taken into account in the interpretation of both tests.

Since immediate alternatives to anti-FXa and aPTT are not routinely available, and we would suggest the use of anti-FXa as the primary UFH monitoring assay rather than aPTT, the use of the latter may lead to administration of inappropriate doses of UFH. Furthermore, anti-FXa and aPTT failed to predict serious bleeding or thrombotic complications. These results imply that these haemostatic complications have multiple causes beyond anticoagulation alone, thus highlighting the complexity of VA-ECMO coagulopathy.

## Figures and Tables

**Table 1:** Characteristics of the study population (*n* = 265).

Characteristics	*N*	Values
Age, years	265	55 ± 14
Body mass index, kg/m^2^	258	27.5 ± 5.7
Male gender	265	183 (69.1)
Comorbidities		
Strokes	264	8 (3.0)
Atrial fibrillation	263	95 (36.1)
Diabetes mellitus	264	61 (23.1)
Hypertension	264	144 (54.5)
Hypercholesterolaemia	252	93 (36.9)
Chronic kidney disease	256	97 (37.9)
P2Y12 inhibitors during ECMO	257	21 (8.2)
Simplified acute physiology score II	264	58.3 ± 22.0
Lactate on admission, mmol/L	176	4.5 (2.4 to 9.5)
Description of ECMO support		
Aetiologies of refractory shocks	265	
Postoperative low cardiac output syndrome		90 (34.0)
Primary graft dysfunction		15 (5.7)
Myocardial infarction		76 (28.7)
Acute on chronic heart disease		40 (15.1)
Pulmonary embolism		9 (3.4)
Myocarditis		16 (6.0)
Poisoning		7 (2.6)
Others		12 (4.5)
Postcardiotomy shock	265	103 (38.9)
Duration of ECMO support, days	265	7 (3 to 11)
Peripheral ECMO	265	263 (99.2)
Left ventricle unloading strategies	265	51 (19.2)
Left ventricle venting		10 (3.8)
Cannulation upgrade		10 (3.8)
Intra-aortic balloon pumping		4 (1.5)
Impella CP/5.0		27 (10.2)
Thrombotic complications	265	87 (32.8)
Description of thrombosis sites **		102
Ischemic stroke		46 (45.1)
Limb ischaemia		15 (14.7)
Cannula/circuit thrombosis		26 (25.5)
Others		15 (14.7)
Serious bleeders	265	150 (56.6)
Description of bleeding sites *		206
Pericardial		79 (38.4)
Cannula		46 (22.3)
Pleural		19 (9.2)
Otorhinolaryngological area		16 (7.8)
Gastrointestinal tract		10 (4.9)
Haemoptysis		9 (4.4)
Intracerebral haemorrhage		6 (2.9)
Others *		21 (10.2)
Number of blood products	265	
Packed red blood cells, units	250	10 (5 to 18)
Fresh frozen plasma, units	169	7 (3 to 11)
Platelet concentrate, units	176	3 (2 to 6)
Weaning categories	265	
Successful weaning		120 (45.5)
Heart transplantation		22 (8.3)
Left or bi-ventricular assist devices		20 (7.6)
Death under VA-ECMO support		103 (38.9)
Outcomes	265	
Intensive care unit length of stay (days)		12 (6 to 25)
Hospital length of stay (days)		18 (8 to 41)
28-day mortality		114 (43.0)
Intensive care unit mortality		126 (47.5)
Hospital Mortality		136 (51.3)

Values are numbers (percentage), mean ± standard deviation or median (interquartile range). ECMO, extracorporeal membrane oxygenation; * The other aetiologies were: Acute pulmonary hypertension (2) and cardiac arrest (2) related to ARDS, Takotsubo cardiomyopathy (2), cardiogenic shock related to pheochromocytoma (1) or following liver transplantation (1), cardiac arrest following anaphylactic shock (1), septic cardiomyopathy (1), cardiogenic shock following transcatheter aortic (1) or mitral (1) valve replacement. ** multiple sites in some patients.

## Data Availability

The anonymised datasets analysed during the current study are available from the corresponding author on a reasonable request and in compliance with French laws.

## Supplementary Materials

The following are available online at https://www.mdpi.com/article/10.3390/jcm10102158/s1, Figure S1: Study flowchart. Figure S2: Time courses of reference variables that modulate anti-FXa and aPTT association. Figure S3: Cumulative incidence of serious bleeding and thrombotic complications during ECMO support. Figure S4: Association of anti-FXa and aPTT with serious bleeding during VA-ECMO support. Table S1: Description of technical characteristics of the studied biological variables.
