# Supplementary material for: Evaluation of Anti-Activated Factor X Activity and Activated Partial Thromboplastin Time Relations and Their Association with Bleeding and Thrombosis during Veno-Arterial ECMO Support: A Retrospective Study
Source: J Clin Med. 2021 May 17;10(10):2158. doi: 10.3390/jcm10102158 (PMC8156165; doi:10.3390/jcm10102158)
Supplement: Supplementary file 1 [file jcm-10-02158-s001.zip › jcm-1194698-supplementary/Supplementary files_jcm/Anti-Xa_JCM_Rev_Additional Table 1.pdf]

Additional Table S1. Description of technical characteristics of the studied biological variables.

Variables	Analyzer	Technics and reagents	Normal range or reference
aPTT, seconds	STA-R Max, Stago (Asnières, France)	Chronometric method, TriniCLOT aPTT HS, Tcoag (Wicklow, Ireland)	<41; (Reference=31)
PT, seconds	STA-R Max, Stago (Asnières, France)	Chronometric method, Neoplastin R Kit, Stago (Asnières, France)	13.6
Anti-FXa, UI/mL	STA-R Max, Stago (Asnières, France)	Chomogenic method, Biophen Heparin LRT, HYPHEN BioMed (Neuville -sur-Oise, France)	<0.1
Factor V, %	STA-R Max3, Stago (Asnières, France)	Chronometric method, STA-Deficient V, Stago, Neoplastine R, Stago (Asnières, France)	60 to 120
Fibrinogen, g/L	STA-R Max3, Stago (Asnières, France)	Automated Clauss method, STA®-Liquid Fib, Stago (Asnières, France)	2 to 4
Hemoglobin, g/dL	Sysmex XN, Sysmex France (Roissy CDG, France)	Coulter principle (electrical Impedencemetry) and hydrodynamic focusing	12 to 16
Platelets, 10^9/L	Sysmex XN, Sysmex France (Roissy CDG, France)	Coulter principle (electrical Impedencemetry) and hydrodynamic focusing	150 to 400
Total Bilirubin, mg/L	Cobas®, Roche Diagnostics	Diazo-based colorimetric assay, ROCHE Diagnostics (Amlere, Netherlands)	1 to 12
LDH, UI/L	Cobas®, Roche Diagnostics	Enzymatic according to IFCC reference measurement procedures, ROCHE Diagnostics (Amlere, Netherlands)	135 to 214
aPTT, activated partial thromboplastin time; PT, Prothrombin time; Anti-FXa, Anti-factor X activity; IFCC, International Federation of Clinical Chemistry; LDH lactate dehydrogenase. For the haemostasias tests (aPTT, anti-FXa, PT, FV and Fibrinogen), blood was collected on 0.109M trisodium citrate tube (BD Vacutainer®, BD Diagnostics). The haemostasias were performed on platelet-poor plasma with a residual platelet count <10 10^9/L after a double centrifugation at 2500g for 15 min at room temperature. The delivery time from ICU to hematological			

laboratory is shorten to few minutes tanks to an automatized pneumatic transportation system, the time to completion was < 60 min.
