# Supplementary figures and images for: Evaluation of Anti-Activated Factor X Activity and Activated Partial Thromboplastin Time Relations and Their Association with Bleeding and Thrombosis during Veno-Arterial ECMO Support: A Retrospective Study
Source: J Clin Med. 2021 May 17;10(10):2158. doi: 10.3390/jcm10102158 (PMC8156165; doi:10.3390/jcm10102158)

## Slide 1
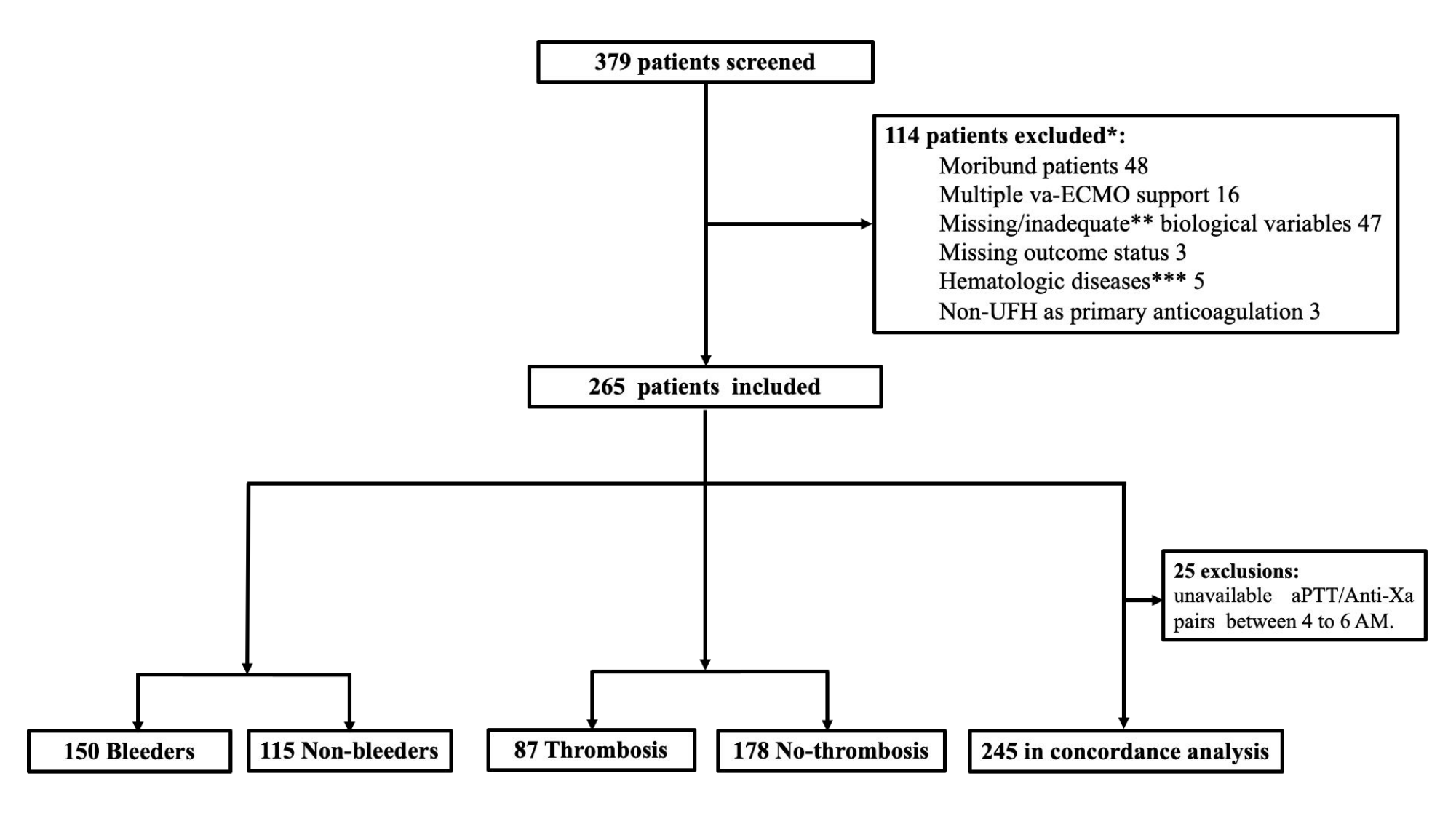

Supplement: Supplementary file 1 [file jcm-10-02158-s001.zip › jcm-1194698-supplementary/Supplementary files_jcm/AntiXa_JCM_Additional_Figure_1.pptx]

## Slide 1
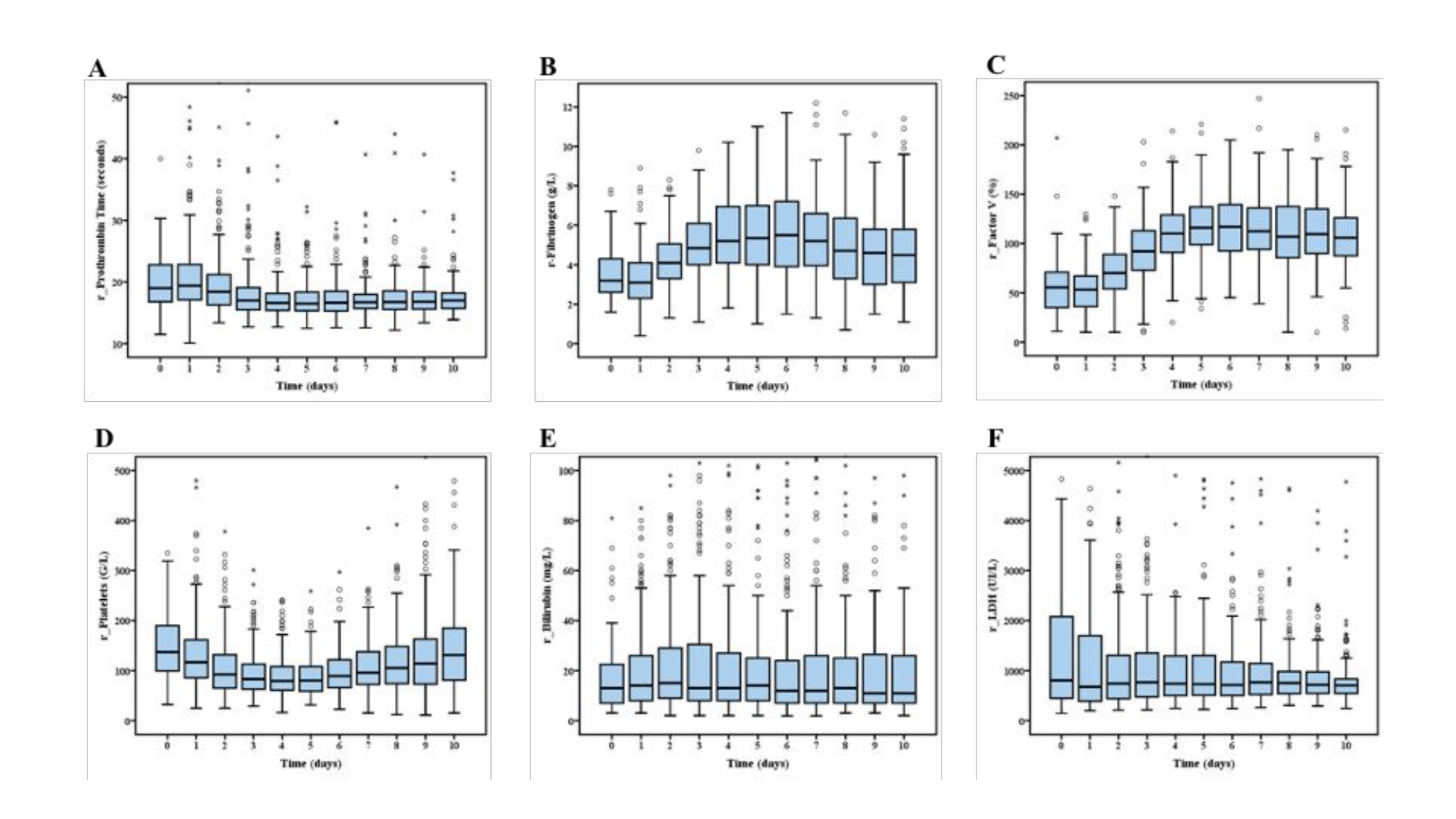

Supplement: Supplementary file 1 [file jcm-10-02158-s001.zip › jcm-1194698-supplementary/Supplementary files_jcm/AntiXa_JCM_Additional_Figure_2.pptx]

## Slide 1
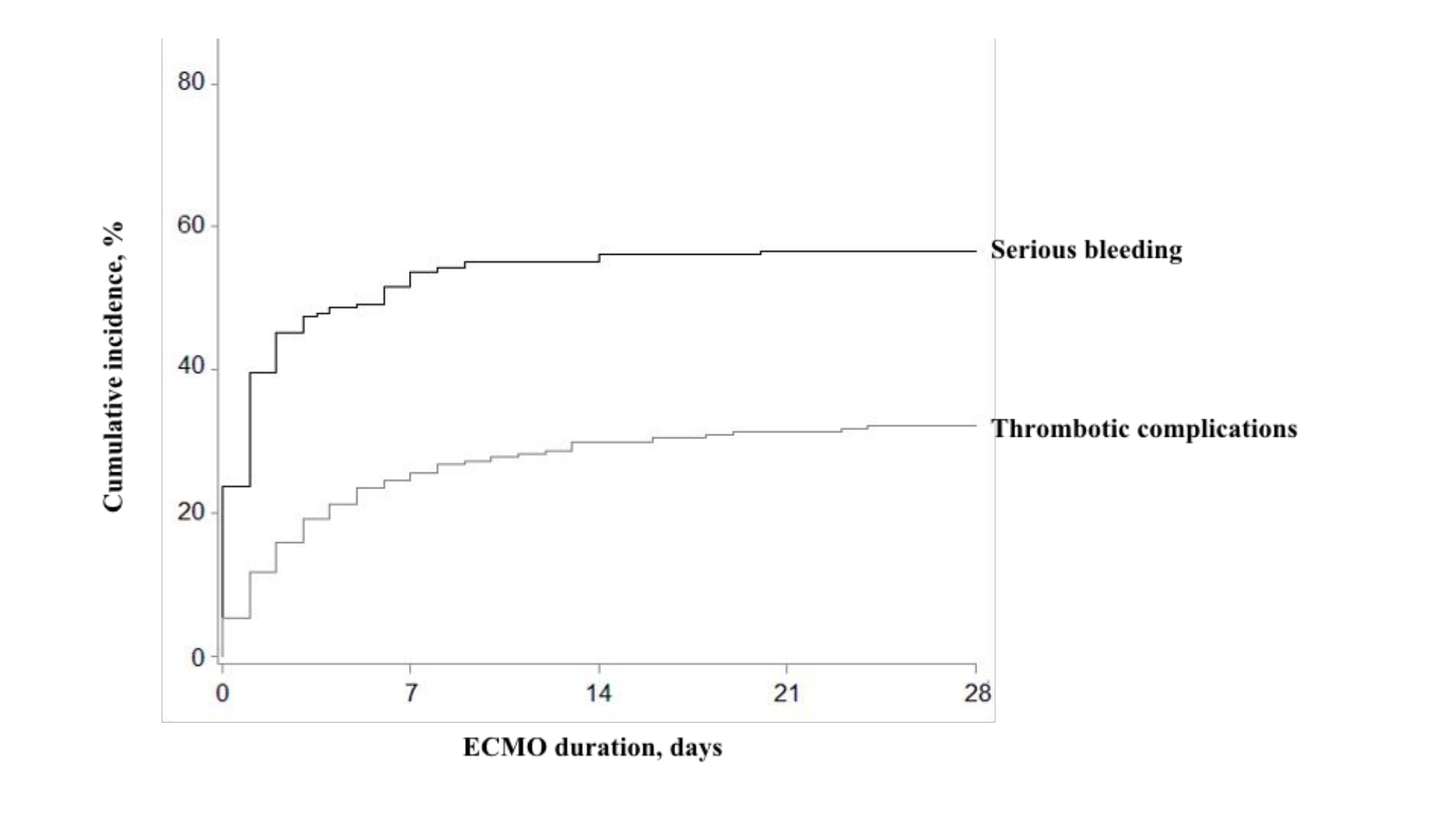

Supplement: Supplementary file 1 [file jcm-10-02158-s001.zip › jcm-1194698-supplementary/Supplementary files_jcm/AntiXa_JCM_Additional_Figure_3.pptx]

## Slide 1
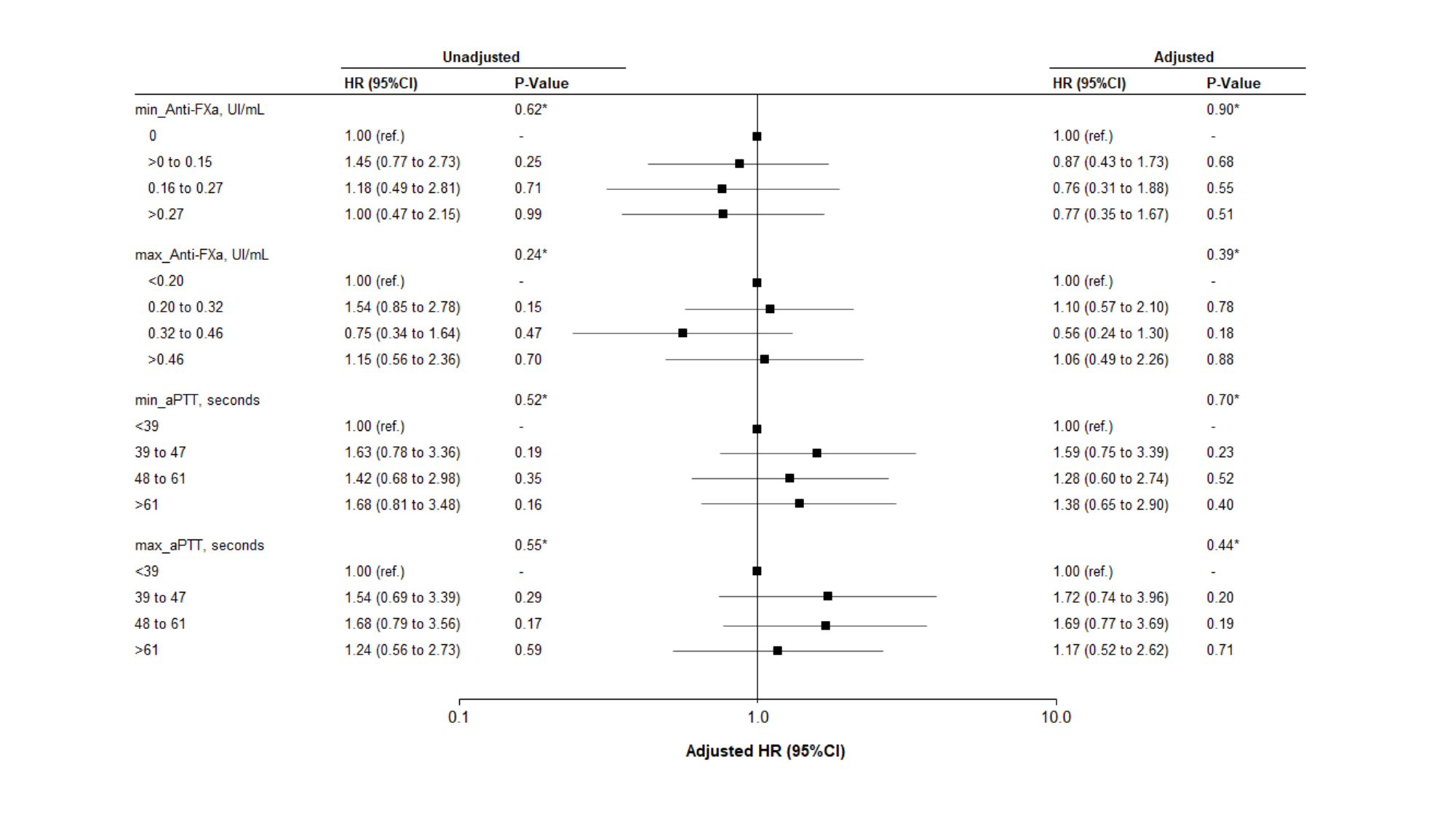

Supplement: Supplementary file 1 [file jcm-10-02158-s001.zip › jcm-1194698-supplementary/Supplementary files_jcm/AntiXa_JCM_Additional_Figure_4.pptx]

## Slide 1
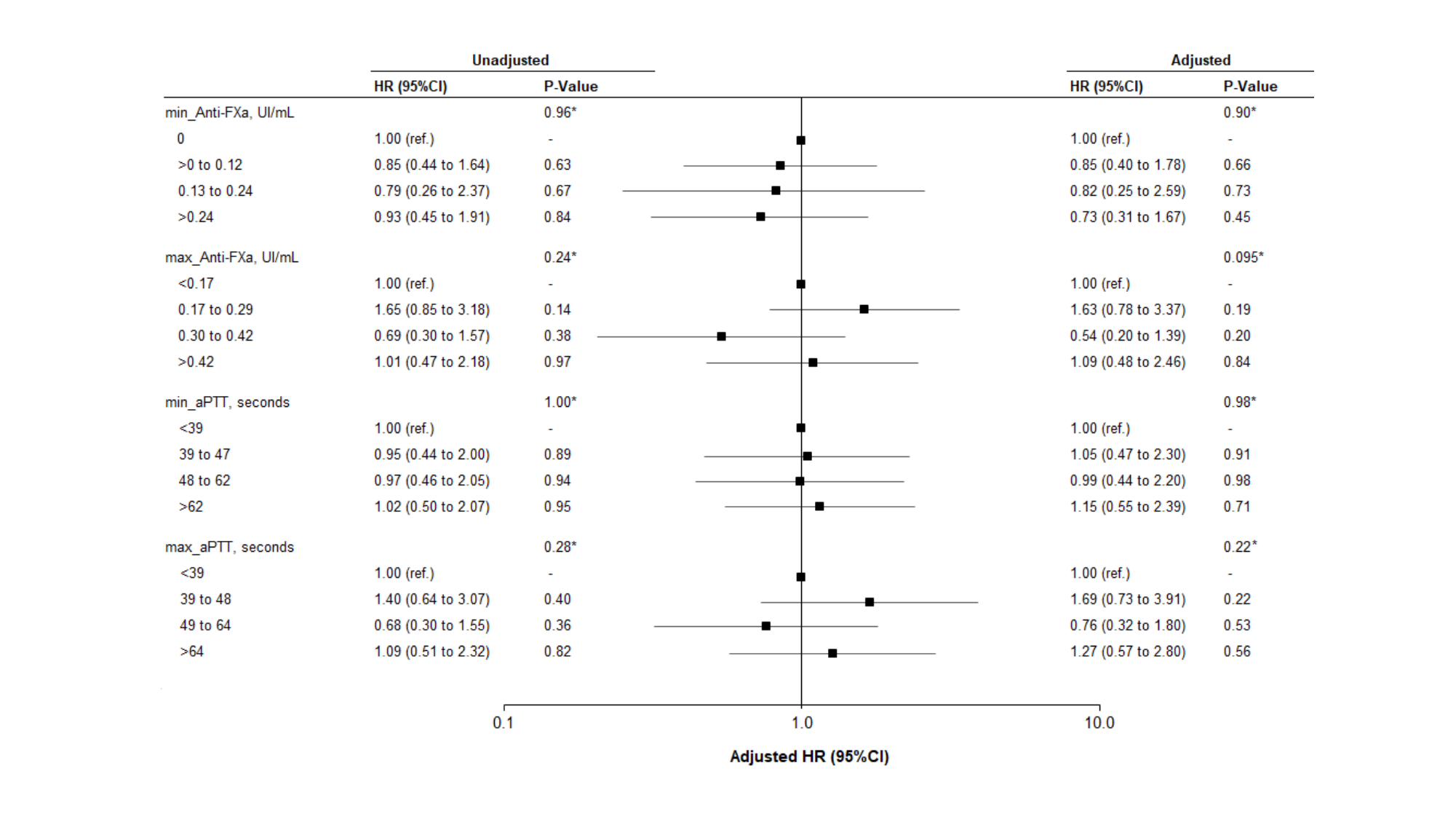

Supplement: Supplementary file 1 [file jcm-10-02158-s001.zip › jcm-1194698-supplementary/Supplementary files_jcm/AntiXa_JCM_Additional_Figure_5.pptx]
